# Identification and Reporting of Patient and Public Partner Authorship on Knowledge Syntheses: Rapid Review

**DOI:** 10.2196/27141

**Published:** 2021-06-10

**Authors:** Ursula Ellis, Vanessa Kitchin, Mathew Vis-Dunbar

**Affiliations:** 1 Woodward Library University of British Columbia Vancouver, BC Canada; 2 University of British Columbia Okanagan Library Kelowna, BC Canada

**Keywords:** PPI, patient and public involvement, coproduction, authorship, systematic review, participatory medicine, patient involvement, patient education, participatory research

## Abstract

**Background:**

Patient and public involvement (PPI) in health research is an area of growing interest. Several studies have examined the use and impact of PPI in knowledge syntheses (systematic, scoping, and related reviews); however, few studies have focused specifically on the patient or public coauthorship of such reviews.

**Objective:**

This study seeks to identify published systematic and scoping reviews coauthored by patient or public partners and examine the characteristics of these coauthored reviews, such as which journals publish them, geographic location of research teams, and terms used to describe patient or public partner authors in affiliations, abstracts, or article text.

**Methods:**

We searched CAB Direct, CINAHL, Cochrane Database of Systematic Reviews (Ovid), Embase (Ovid), MEDLINE (Ovid), and PsycInfo from 2011 to May 2019, with a supplementary search of several PPI-focused databases. We refined the Ovid MEDLINE search by examining frequently used words and phrases in relevant search results and searched Ovid MEDLINE using the modified search strategy in June 2020.

**Results:**

We screened 13,998 results and found 37 studies that met our inclusion criteria. In line with other PPI research, we found that a wide range of terms were used for patient and public authors in author affiliations. In some cases, partners were easy to identify with titles such as patient, caregiver or consumer representative, patient partner, expert by experience, citizen researcher, or public contributor. In 11% (n=4) of studies, they were identified as members of a panel or advisory council. In 27% (n=10) of articles, it was either impossible or difficult to tell whether an author was a partner solely from the affiliation, and confirmation was found elsewhere in the article. We also investigated where in the reviews the partner coauthors’ roles were described, and when possible, what their specific roles were. Often, there was little or no information about which review tasks the partner coauthors contributed to. Furthermore, only 14% (5/37) of reviews mentioned patient or public involvement as authors in the abstract; involvement was often only indicated in the author affiliation field or in the review text (most often in the methods or contributions section).

**Conclusions:**

Our findings add to the evidence that searching for coproduced research is difficult because of the diversity of terms used to describe patient and public partners, and the lack of consistent, detailed reporting about PPI. For better discoverability, we recommend ensuring that patient and public authorships are indicated in commonly searched database fields. When patient and public-authored research is easier to find, its impact will be easier to measure.

## Introduction

### Background

Patient and public involvement (PPI) in health is an area of growing research interest. As this interest has increased, the need to effectively report on patient and public contributions to the research process has also increased. Reporting on PPI has several benefits, including the ability to identify, collate, and understand how such partnerships are undertaken and their impact on the research evaluated [1]. As PPI improves research quality and relevance, identifying studies that integrate PPI is important for practice and policy [2]. Considering this, we investigated the degree to which systematic, scoping, and related reviews identify patient and public partners as coauthors.

Multiple frameworks have been proposed to improve the reporting of patients’ roles and levels of involvement in research [3-9]. Notably, in primary research, the GRIPP (Guidance for Reporting Involvement of Patients and Public) checklist was developed in 2011 [2]. A revision, GRIPP2, published in 2019, introduced short and long forms of the checklist [1]. These checklists guide authors to report on the methods used for PPI and the results and impacts of PPI in a study. In synthesis research, the ACTIVE (Authors and Consumers Together Impacting on Evidence) framework provides reporting guidance specifically for systematic reviews [10]. The framework’s continuum of involvement breaks the systematic review process into 12 stages and describes 5 levels at which patients or the public can be involved at each stage: leading, controlling, influencing, contributing, and receiving.

Despite the development of these frameworks, the identification of PPI remains problematic. One issue is a lack of reporting; a 2019 review by Fergusson et al [11] found that from 2777 screened clinical trials, only 23 reported on patient engagement. The second issue is the lack of guidance on reporting structures that would allow discoverability of such research in databases of published research. Although the GRIPP2 long form suggests that the author supplied keywords “[i]nclude PPI, ‘patient and public involvement,’ or alternative terms” [1], a pervasive issue is the plethora of terms researchers may use to describe PPI [12,13]. These shift geographically, PPI itself being overwhelmingly used in the United Kingdom, whereas research in Australia, Canada, and the United States frequently uses divergent terms [14]. The concept of *participants* can vary widely, including *consumers*, *service users*, *lay people*, *carers* or *caregivers*, and the ambiguous term *stakeholders*, which may represent any number of roles not related to research methodology or implementation.

We were particularly interested in reporting PPI through authorship; an important aspect of PPI is the inclusion and recognition of contributions to research outputs. Neither GRIPP nor GRIPP2 address best practices on when and how to include patient and public partners as coauthors in primary research. Although the ACTIVE framework identifies “writing and publishing the review” as one stage of potential involvement, it lacks specific guidance on including partners as coauthors. In a recent systematic review, Arnstein et al [15] presented a set of 21 recommended best practices for involving patient partners as coauthors in health research; one recommendation is “[d]ocument, in the manuscript, the involvement and role of patient authors (i.e. identify which authors are patients [e.g. Author Affiliation section] and describe their authorship contributions [e.g. Contributorship section]).” In addition to these recommendations, they developed two versions of a patient authorship experience tool to assess the impact and quality of patient involvement.

Synthesis research (systematic and scoping reviews) frequently informs policy, guidelines, and point-of-care tools as well as first-line consultation tools used by practitioners. The prevalence and impact of PPI in systematic reviews have been the subject of many studies [16-25]. Evidence synthesis bodies have taken up the call to enhance use and reporting of PPI in reviews—Cochrane launched the ACTIVE project to encourage reviewers to meaningfully engage patients and the public in creating reviews [10,26], whereas in environmental research, the Stakeholder Engagement in Evidence Synthesis website hosts a plethora of resources on involving the public in reviews [27]. Identification of participation through authorship can clearly signal the integration of PPI in the synthesis process.

Our inquiry, to identify systematic and scoping reviews coauthored by patient and public partners, hoped to inform how, and how frequently, authorship in syntheses is being attributed. Our inquiry was informed by the following research question:

Among published systematic and scoping reviews, either on the topic of PPI or including PPI more generally, are the patient and public partners included as coauthors? If so, how are these studies identified and indexed?

### Objectives

Our process is guided by the following objectives:

1. Identify published systematic and scoping reviews coauthored by patient or public partners.
2. Identify if reviews in certain journals, countries, or disciplines are more likely to include patient or public partner authors.
3. Determine useful search terms to find reviews with patient or public partner authors, based on how such authors are described in affiliations, abstracts, or article texts.

## Methods

### Registration and Eligibility Criteria

We registered our protocol on OSF on August 23, 2019; the protocol and other supplementary materials for this review are available on OSF [28]. We defined our eligibility criteria as illustrated in Textbox 1.

**Inclusion and exclusion criteria.**

**Inclusion Criteria**

- Systematic or scoping reviews on health topics that state that at least one author is a patient or public partner
- Anything that self-identifies as, or employs methodologies used in, a comprehensive review of the literature
- Published since 2011
- Must include a nonacademic partner
- Full text available in English

**Exclusion Criteria**

- Protocols
- Conference abstracts
- Reports on trials
- Case studies on patient engagement
- Where the patient or public partner has an academic title or affiliation

In interpreting PPI in papers that did not employ this specific terminology, we used National Institute for Health Research (NIHR) INVOLVE’s definition of patient and public partners, as expressed in the study by Boote et al [16]. INVOLVE defines the public as “patients and potential patients; people who use health and social services; informal carers; parents or guardians; disabled people; members of the public who are potential recipients of health promotion programmes, public health programmes and social service interventions; organisations that represent people who use services.” INVOLVE defines public involvement in research as “doing research ‘with’ or ‘by’ the public, rather than ‘to’, ‘about’ or ‘for’ the public.”

We limited to systematic reviews, scoping reviews, or reviews employing recognized methodologies employed in these review types, published after 2011, aligning with the publication of GRIPP, the first published reporting guidelines on reporting on PPI [2].

### Study Selection

Our search strategy was based on the validated filter for PPI published by Rogers et al [12]. This was further supplemented by terms derived from an analysis of 80 primary research articles on partnership research, derived from a previous survey of review articles [14]. Finally, terms identified by a canvas of previously published reviews on the subject were iteratively collected and compared against those from the above two sources. To limit to systematic and scoping reviews, we used the terms in PubMed’s systematic review filter plus some additional terms for scoping reviews or other knowledge syntheses [29]. A librarian unaffiliated with the project peer reviewed the Ovid MEDLINE search strategy using the Peer Review of Electronic Search Strategies checklist [30]. Multimedia Appendix 1 displays the initial Ovid MEDLINE search strategy.

Searches were run in 6 databases from 2011 to May 23, 2019: CAB Direct, CINAHL, Cochrane Database of Systematic Reviews (Ovid), Embase (Ovid), MEDLINE (Ovid), and PsycInfo (EBSCO).

Several additional sources were hand searched in August and September 2019:

- Patient-Centered Outcomes Research Institute Engagement in Health Research Literature Explorer
- Patient-Centered Outcomes Research Institute in the Literature
- NIHR INVOLVE Publications Library and Evidence Library
- The Evidence for Policy and Practice Information and Co-ordinating-Centre systematic reviews
- Patient Experience Journal
- Journal of Participatory Medicine
- Centre of Excellence on Partnership with Patients and the Public
- McMaster University Public and Patient Engagement Collaborative

In addition, we reviewed the reference lists and studies included in other systematic reviews to identify further studies. 

With the initial search results from May 2019, all 3 authors screened a sample of 100 titles and abstracts to determine interrater agreement; with 82% (82/100) consensus between all reviewers, we then split the results into 3 segments for title and abstract screening. One author screened, with a second author deciding on studies that were labeled unsure. At this stage, we included or noted reviews that either stated they incorporated PPI or were on topics that would likely involve patients as unsure, excluding reviews about preclinical or other research that does not lend itself to inclusion of patient expertise.

At the full-text screening stage for the initial search results, articles were divided into 3 segments, with one reviewer first screening for any studies that could clearly be excluded. We looked at author affiliations, methods, author contributions, and acknowledgment sections for indicators that one or more authors met our definition of a patient or public partner. All 3 authors then assessed all reviews marked as include or unsure; in cases of disagreement, we discussed reaching a consensus. Some relevant articles may have been excluded at this stage because the partner author was not explicitly identified as required by our inclusion criteria.

The Ovid MEDLINE search was updated on June 8, 2020, using a modified search strategy. The new strategy was developed by examining the frequency of terms used in the titles and abstracts of the 953 articles that reached the initial full-text screening stage. We used R to extract the n-grams from the titles and abstracts of the 953 articles [31]. We then reviewed the most common n-grams and discussed which n-gram should be included in the modified version of the search. This process yielded new search terms for both the patient and public partner concept and the systematic review concept. The full modified Ovid MEDLINE search strategy is available in Multimedia Appendix 2, and the R code for extracting the n-grams is available in OSF [28]. After removing duplicates, 1 author screened titles and abstracts, and 2 authors screened each full-text article; all 3 authors discussed articles marked as include or unsure to reach agreement.

### Data Extraction

Included reviews were divided into 3 groups, and each author extracted data from reviews in 1 group. The areas of ambiguity in data extraction were discussed by all 3 reviewers.

We extracted data about author affiliation of patient or public partners, journal, country of partners, how they have contributed to reviews, and in what sections of the reviews these contributions were described. Where sufficient information was available, we coded the partners’ roles in line with the 12 stages of a systematic review as outlined in the ACTIVE framework [10]: (1) develop question, (2) plan methods, (3) write and publish protocol, (4) develop search, (5) run search, (6) select studies, (7) collect data, (8) assess risk of bias, (9) analyze data, (10) interpret findings, (11) write and publish review, and (12) knowledge translation and impact.

## Results

### Search Results and Screening

The initial database search in May 2019 returned 25,853 results, with an additional 35 results identified through other means such as cited reference searching; 13,958 results remained after deduplication. A total of 953 reviews were screened in the full text. Preliminary findings of our research, presenting the results of our initial database and supplementary hand search, were presented in a poster at the 26th Cochrane Colloquium [32].

**Figure 1:**
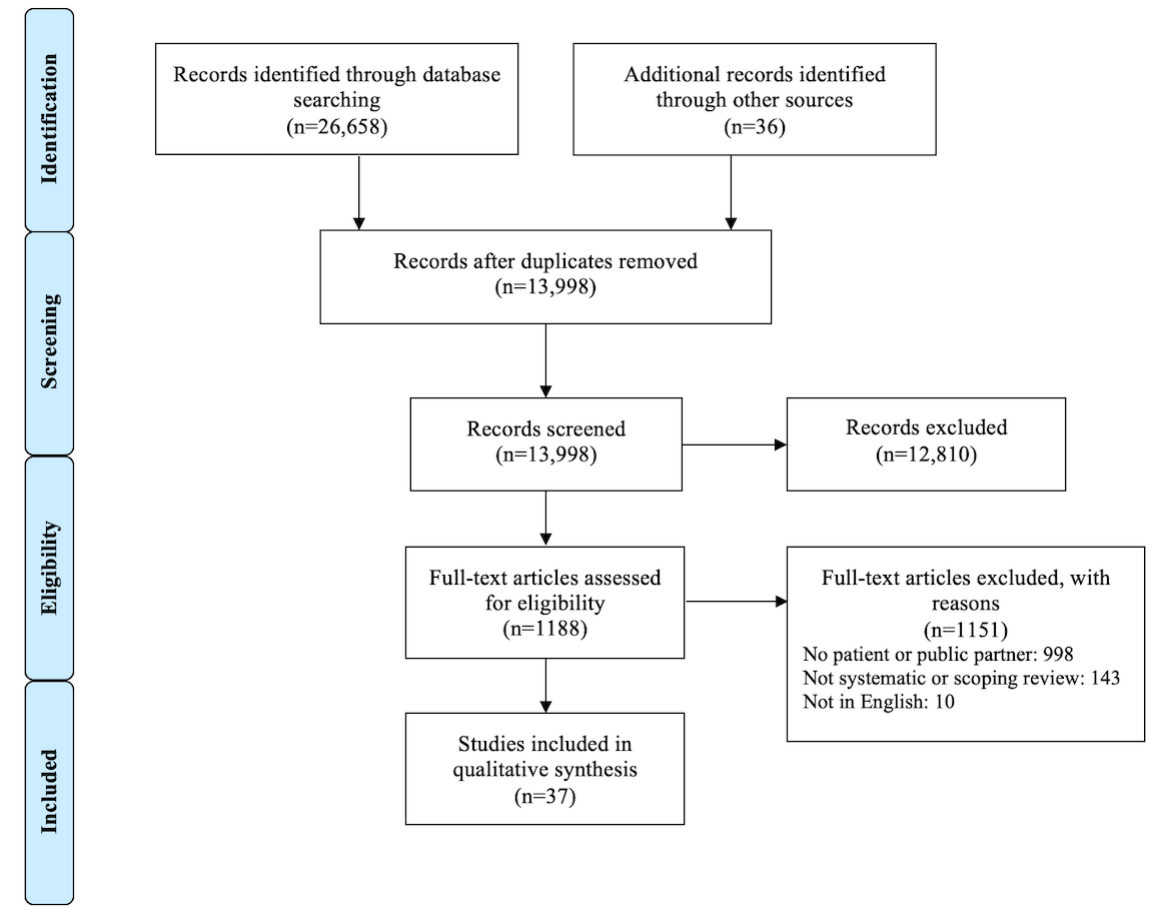
PRISMA (Preferred Reporting Items for Systematic Reviews and Meta-Analyses) flow diagram.

An additional 805 results were found by the modified updated Ovid MEDLINE search run on June 8, 2020. One additional study was identified for inclusion because it was mentioned on social media after the search update was run, bringing the total number of studies identified through other means to 36. The PRISMA (Preferred Reporting Items for Systematic Reviews and Meta-Analyses) flow diagram presents the total number of search results, including the June 2020 update (Figure 1). A total of 37 articles were included in our overview. 

### Characteristics of Included Studies

The characteristics of the 37 included studies are summarized in Table 1. Reviews were published across 28 journals representing a range of health disciplines. Many terms were used to describe the patients and public partners in author affiliations. In some cases, partners were easy to identify with titles such as patient, caregiver or consumer representative, patient partner, expert by experience, citizen researcher, or public contributor. In others, they were identified as members of a panel or advisory council. Some studies identified *partner* as members of a panel or advisory group; in 11% (n=4) of the articles, a panel or other body was named as an author rather than individual contributors [14,33-35]. Finally, in 27% (n=10) of articles, it was either impossible or difficult to tell whether an author was a partner solely from the affiliation, and confirmation was found elsewhere in the article.

The majority (21/37, 57%) of reviews had patients or public partners based in the United Kingdom (Table 2).

**Figure 2:**
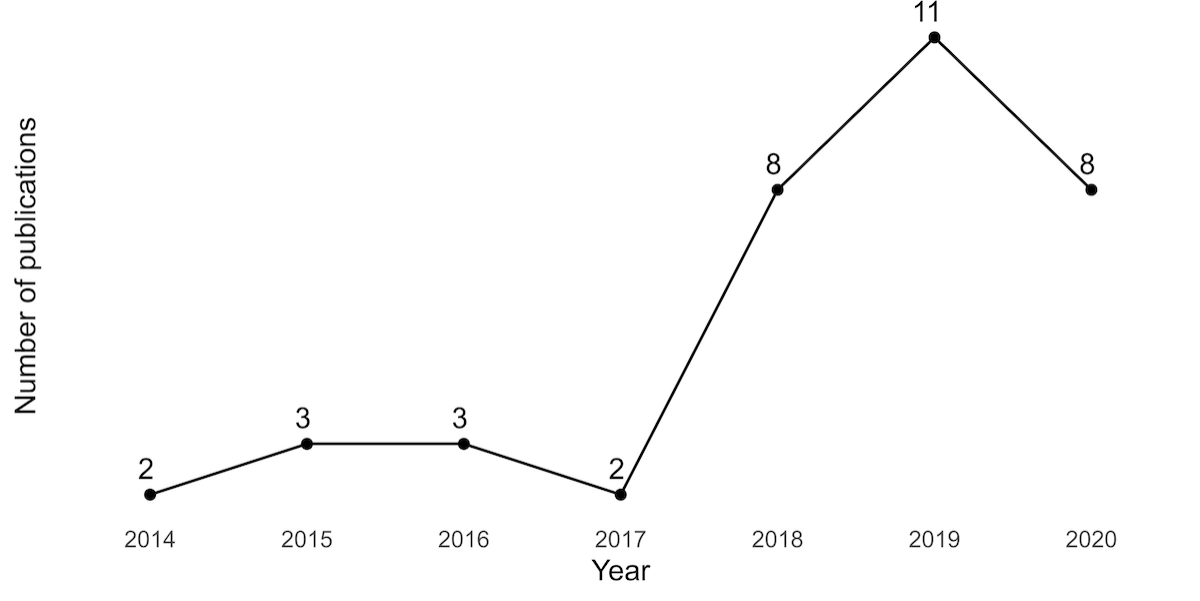
Number of reviews with a patient or public partner coauthor by year.

The number of reviews increased notably from 2018 onward (Figure 2). 

**Table 1:** Selected characteristics of included studies.

Study	Author affiliation of patient or public partner	Journal
Brett et al [36]	UK Clinical Research Collaboration; University/Users Teaching and Research Action Partnership	The Patient
Brett et al [37]	UK Clinical Research Collaboration; University/Users Teaching and Research Action Partnership	Health Expectations
Aslakson et al [38]	Patient/family member coinvestigator, Architecture by Design	Journal of Comparative Effectiveness Research
Jones et al [39]	Patient representative, Colon Aid PPI^a^ Group, Yeovil District Hospital Foundation Trust	Annals of Surgery
Whitton et al [40]	Cochrane Skin Group	Cochrane Database of Systematic Reviews
Garvelink et al [41]	Caregiver representative in the Population Health and Practice-Changing Research Group of the Research Centre, CHU de Québec	Health Affairs
Morley et al [20]	Consumer representative, Cochrane Pregnancy and Childbirth	Research Involvement and Engagement
Souleymanov et al [42]	Harm Reduction Peer Street Outreach Coordinator, Queen West Central Toronto Community Health Centre	BMC Medical Ethics
Clarkson et al [33]	Members of the HoSt‐D^b^ Programme Management Group	Journal of Advanced Nursing
Kronenberg et al [43]	Expert by experience	The British Journal of General Practice
Bethell et al [44]	Ontario Dementia Advisory Group	Dementia
Crocker et al [45]	National Institute for Health Research Oxford Biomedical Research Centre	BMJ
Evans et al [34]	North Bristol Microbiology Patient Panel	Health Expectations
Fergusson et al [11]	Patient Partner, SPOR^c^ National Steering Committee	Research Involvement and Engagement
Jennings et al [46]	RECOLLECT Lived Experience Advisory Panel	BMC Psychiatry
Jorgensen et al [47]	Patient and Public Representative	Qualitative Health Research
Pollock et al [48]	None, just location	Systematic Reviews
Price et al [49]	Citizen Researcher	Journal of Evaluation in Clinical Practice
Baines et al [50]	Volunteer Mental Health Patient-Research-Partner	Journal of Health Services Research & Policy
Evans et al [51]	Mojatu Foundation	BMJ Open
Gonzalez et al [52]	Patient Representative, Federal Joint Committee, Gemeinsamer Bundesausschuss	BMJ Open
Greenhalgh et al [4]	Patient Adviser, Nuffield Department of Primary Care Health Sciences, University of Oxford	Health Expectations
McCarron et al [53]	Patient coinvestigators, Community Health Sciences, University of Calgary	Systematic Reviews
McGrath et al [54]	Public contributor	The International Journal on Drug Policy
Moore et al [55]	Biomedical Research Centre Patient & Public Involvement Group, University College London Hospitals	Health Technology Assessment
Oldfield et al [56]	General Patient and Family Advisory Council, Yale-New Haven Hospital	Journal of General Internal Medicine
Planner et al [57]	NIHR^d^ School for Primary Care Research	Trials
Scholz et al [58]	Consumer representative	Palliative Medicine
Sherriff et al [35]	Health4LGTBI Network	Health Expectations
Bird et al [59]	Patient Partner, McMaster University	Health Expectations
Brush et al [60]	Friends of Parkside	Health Education & Behavior
Gordon et al [61]	COMENSUS^e^ Group	Medical Teacher
Graham et al [62]	Radcliffe Women’s Health Patient and Public Participation Panel	BJOG
Hoekstra et al [14]	SCI^f^ Guiding Principles Consensus Panel	Health Research Policy and Systems
Hung et al [63]	Community Engagement Advisory Network	Dementia
Maidment et al [64]	PPI representative	BMC Geriatrics
Arnstein et al [15]	Consumer Forum, National Cancer Research Institute; Research Involvement and Engagement; International Alliance of Patients’ Organizations	Research Involvement and Engagement

^a^PPI: patient and public involvement.

^b^HoST-D: Home Support in Dementia.

^c^SPOR: strategy for patient-oriented research.

^d^NIHR: National Institute for Health Research.

^e^COMENSUS: Community Engagement and Service User Support.

^f^SCI: spinal cord injury.

**Table 2:** Country of patient and public partner authors.

Country	Review, n (%)
United Kingdom	21 (57)
Canada	8 (22)
United States of America	3 (8)
Unknown or multi-state	2 (5)
Germany	1 (3)
Denmark	1 (3)
Australia	1 (3)

In many reviews, little information was provided about the specific roles of patients or public partners in the review (Table 3). In 24% (9/37) of the reviews, the author affiliation field was the only place in the paper indicating the involvement of a patient or public partner; these reviews are identified in Multimedia Appendix 3. For reviews that described the partners’ contributions in the text, the most common location of this report was in the methods section (18/37, 49%). Only 14% (5/37) of reviews articulated patient and public contributions in a field routinely searched in databases of journal literature, the abstract.

**Table 3:** Sections of review besides author affiliation field indicating patient or public partner involvement.

Section	Review, n (%)
Methods	18 (49)
Contributions	9 (24)
Discussion	5 (14)
Abstract	5 (14)
Results	3 (8)
Introduction	2 (5)
Acknowledgments	2 (5)
Patient, service user or end user involvement	2 (5)
Limitations	1 (3)
Appendix	1 (3)

We coded patient and public partners’ roles in reviews in alignment with the 12 review stages identified in the ACTIVE framework (Table 4). In 49% (18/37) of cases, insufficient detail was available on the stages of the review that partners contributed to, and the authors coded these as *unclear*. More detailed information showing the location of reporting and specific partner roles for each included review is available in Multimedia Appendix 3.

**Table 4:** Patient involvement in the 12 review stages identified by the ACTIVE (Authors and Consumers Together Impacting on Evidence) framework.

Stage	Review, n (%)
Unclear	18 (49)
Develop question	8 (22)
Plan methods	4 (11)
Write and publish protocol	4 (11)
Develop search	6 (16)
Select studies	7 (19)
Collect data	4 (11)
Analyze data	9 (24)
Interpret findings	10 (27)
Write and publish review	12 (32)
Knowledge translation and impact	6 (16)

## Discussion

### Lack of Clarity in Reporting Public and Patient Partner Roles

There are many potential capacities in which PPI can take shape and many stages of the research cycle in which that PPI may be implemented. However, our final set of papers included a subset for which there was a lack of clarity in the terminology used to report and describe public and patient partner involvement, making it a challenge to classify partners’ roles. The nuances surrounding the extent to which patient and public coauthors contribute to research vary and reflect their educational and experiential backgrounds.

This subset of papers used vague or nonspecific language to describe both roles and contributions. Three instances include Clarkson et al [33], Bethell et al [44], and Evans et al [34]. Clarkson et al [33] provide attribution in the acknowledgments section with a short description where the authors “thank...our Patient, Public and Carer Involvement (PPCI) group for their comments from the synthesis.” The comments referred to are not concretely linked to the Patient, Public and Carer Involvement group in the manuscript. In Bethell et al [44], a scoping review looking at dementia care, the partner role is described in a dedicated section, “Engagement of persons with dementia in the research process” as “[t]wo people with dementia, working with the Ontario Dementia Advisory Group (ODAG), were involved in the execution and translation phases of this project.” Although useful to have a clearly delineated section to describe the partnership, it is unclear what *execution* means. Finally, in Evans et al [34], it is almost impossible to decipher the actual contributions of the partner from the authors’ indeterminate description: “panel members were invited to contribute to shaping the discussion section.” Although this describes participation, it does not describe contribution nor are actions like *invited* to and *shape* measurable.

Two instances in which the role of the patient or public partner was unclear include Price et al [49] and Baines et al [50]. In the study by Price et al [49], it is difficult to determine which tasks were completed by a coauthor and which were completed by volunteers because the narrative reports the 2 together. In the section “Our PPI for this Systematic Overview,” although roles are clearly described, their assignment was unclear, making it impossible to distinguish between those activities completed by a volunteer from the Cochrane Task Exchange and those from the 3 volunteers from Empower. In Baines et al [50], although the authors describe a collaboration, “[a]ll research was conducted in collaboration with a volunteer mental health patient research partner who has extensive experience of receiving psychiatric care. Published principles of PPI were followed to support this involvement.” the role and contributions of this collaboration are not described.

Patient and partner expertise may impact the research and summary process in many ways; articulating the roles and contributions of these partners in clear and measurable language allows the reader to evaluate the strengths and limitations of a given study and its methodological rigor. The more closely aligned these descriptions are with an explicitly defined taxonomy, the easier is their interpretation. Our study focused on publications after the introduction of GRIPP in 2011; we wondered if the introduction of GRIPP for reporting on PPI would result in an increase in patient and public partner coauthored reviews, and more detailed descriptions of the roles of these coauthors. Although the number of studies we identified increased over the period 2011-2020, only one review, Arnstein et al [15], used a framework to report their PPI methods.

### Location of Reporting

One challenge of this study was identifying patient and public coauthored secondary research. Reproducible systematic and scoping reviews that contribute to the evidence base rely on abstracting and indexing databases that permit a search to be fully replicable irrespective of the computing environment used. Consequently, the discoverability of PPI contributions needs to be reported at a level captured by these indexing services. In general, titles, abstracts, and author-supplied keyword fields are the primary fields queried, whereas author affiliations can sometimes be queried, and some indexing services include additional controlled vocabulary fields to aid in discoverability. These controlled vocabularies often also capture study methods or publication types.

This challenge of discoverability is highlighted in our own findings, where only 14% (5/37) of the identified articles articulated patient and public contributions in a commonly indexed field, the abstract. The benefits of reporting PPI are limited if PPI cannot be readily identified in systems designed to index and access this research. We are certain that we did not identify all patient and public coauthored systematically conducted secondary research articles in the searched databases (in addition to reporting issues mentioned here, see *Limitations*), and this primarily suggests that not only does reporting need to be better but better guidance is required on where reporting should be done. Supplementation by controlled vocabularies or publication types would further bolster these efforts.

### Authorship Versus Acknowledgment

During full-text screening, we encountered many reviews that acknowledged significant contributions from patient or public partners but named no partners as coauthors. This is consistent with the findings of other publications in participatory research [65]. Recognizing partners as authors indicates that they had substantial involvement in the research; however, partners may not accept or receive authorship for various reasons. For instance, the authors may want to preserve their anonymity, as was noted in the review by Sherriff et al [35], where the coauthor was a collective entity (Health4LGBTI Network) and individuals were not named. Furthermore, many health journals require authors to meet the 4 ICMJE criteria, and some partners may be unable or unwilling to fulfill all of these criteria [66,67]. 

### Strengths and Limitations

#### Strengths

Our study adds to the literature on PPI in knowledge syntheses by collating 37 examples of reviews with patient and public coauthors; to our knowledge, no other study has identified as many instances of patient or public systematic or scoping review coauthors. Our initial search terms were sensitive; we searched numerous databases and other sources and screened nearly 14,000 unique search results. Our approach to updating our search by reviewing frequently used terms from the initial round of search results may be a useful technique for other researchers to adopt. Furthermore, the search terms we developed may help other researchers locate patient and public-authored research.

The data we extracted about author affiliations adds to existing evidence about the diversity of terms used to describe patients and public partners. Our extraction of where in reviews coauthor roles are described, as well as which review tasks they contributed to, provides insight on where current reporting practices are lacking.

#### Limitations

We limited our search to terms related to PPI and post-2011 publications in English because it was not feasible for us to screen all systematic and scoping reviews. These limitations may have introduced a bias. One flaw we discovered with our initial search strategy is that use of the string (patient* adj3 involv*) found many reviews that simply reported that the included studies “involved n patients,” with no actual PPI content.

During title and abstract screening, we limited inclusion to articles that either explicitly talked about PPI or described behavioral or lifestyle interventions, in line with the findings of Wale et al [68] about topics that are likely to engage patients. At the full-text screening stage, it was often impossible to verify that an author was a patient or public partner from the review itself. Furthermore, patients may have multiple roles—as a researcher and as a person with lived experience—or professional roles as patient representatives. Although these articles may have had patient or public authorship, a lack of detailed reporting made verification indeterminable.

This study focused on systematic, scoping, and other reviews that met the minimum criteria for knowledge synthesis: searching more than one database, reporting at least one reproducible search strategy, and reporting the total number of results found and screened. Studies that used multiple methodologies in conjunction with some form of systematic search of the literature; studies that derived findings from other qualitative methods, for example, as consensus methods such as Delphi studies, were excluded. Therefore, we cannot suggest that our findings reflect all derivatives of systematic approaches to secondary research.

### Conclusions

For PPI research to be more fully used and its benefits realized, reporting of this research should be undertaken in such a way that allows for clear identification, which then permits discovery and retrieval. Although reporting frameworks and checklists exist to help guide researchers in both original and synthesis research, they are not harmonized with the current structure of the discovery tools—bibliographic databases—used in the search and retrieval of original research. This makes systematic discovery and retrieval of PPI research—and in particular PPI coauthored research—a challenge, as evidenced by this study. In fact, the methods used to run our updated search strategy identified novel language used to describe both PPI and synthesis literature.

Our findings support previous research that suggests enhanced PPI reporting in systematic reviews allows for better interpretation of the study’s design and results. Our findings also suggest that changes are needed to support the discovery of this research through bibliographic databases. This latter issue represents a point of potential collaboration between authors through enhanced reporting, publishers through encouragement to authors to report on these methodological approaches, and database providers, through added metadata fields to collate this research. One example of a database incorporating added metadata is NIHR INVOLVE’s Evidence Library, which indicates whether patients or caregivers are authors of each included article. Such changes will make patient-authored research easier to identify in databases. When this research is easier to find, its impact will also be easier to measure.

## Appendix

Multimedia Appendix 1 Initial search strategy for Ovid MEDLINE and Epub ahead of print; in-process; and other nonindexed citations, daily, and versions (May 23, 2019).

Multimedia Appendix 2 Modified search strategy for Ovid MEDLINE and Epub ahead of print; in-process; and other nonindexed citations, daily, and versions (run June 8, 2020).

Multimedia Appendix 3 Location of reporting about patients and public partners and their roles when specified. When insufficient information was provided to connect the patient or partner role with any of the 12 review stages in the ACTIVE (Authors and Consumers Together Impacting on Evidence) framework, the authors have noted unclear.

## Abbreviations

ACTIVE: Authors and Consumers Together Impacting on Evidence
GRIPP: Guidance for Reporting Involvement of Patients and Public
NIHR: National Institute for Health Research
PPI: patient and public involvement
PRISMA: Preferred Reporting Items for Systematic Reviews and Meta-Analyses
