# Supplementary material for: Identification and Reporting of Patient and Public Partner Authorship on Knowledge Syntheses: Rapid Review
Source: J Particip Med. 2021 Jun 10;13(2):e27141. doi: 10.2196/27141 (PMC8235296; doi:10.2196/27141)
Supplement: Multimedia Appendix 1 [file jopm_v13i2e27141_app1.docx]

**Multimedia Appendix 1**

Initial search strategy for Ovid MEDLINE(R) and Epub Ahead of Print, In-Process & Other Non-Indexed Citations, Daily and Versions (run May 23, 2019)

| 1 | (patient* adj3 (participat* or involv* or engag* or partner* or collaborat* or contribut* or author* or coauthor* or co-author* or written or cowritten or co-written or coresearch* or co-research* or cocreat* or co-creat* or co-produc* or coproduc* or co-design* or codesign* or input)).tw,kf | 128752 |
| --- | --- | --- |
| 2 | (public adj3 (participat* or involv* or engag* or partner* or collaborat* or contribut* or author* or coauthor* or co-author* or written or cowritten or co-written or coresearch* or co-research* or cocreat* or co-creat* or co-produc* or coproduc* or co-design* or codesign*)).tw,kf. | 14097 |
| 3 | (lay adj3 (participat* or involv* or engag* or partner* or collaborat* or contribut* or author* or coauthor* or co-author* or written or cowritten or co-written or coresearch* or co-research* or cocreat* or co-creat* or co-produc* or coproduc* or co-design* or codesign* or input)).tw,kf | 513 |
| 4 | (people adj3 (participat* or involv* or engag* or partner* or collaborat* or contribut* or author* or coauthor* or co-author* or written or cowritten or co-written or coresearch* or co-research* or cocreat* or co-creat* or co-produc* or coproduc* or co-design* or codesign* or input)).tw,kf | 10286 |
| 5 | (consumer* adj3 (participat* or involv* or engag* or partner* or collaborat* or contribut* or author* or coauthor* or co-author* or written or cowritten or co-written or coresearch* or co-research* or cocreat* or co-creat* or co-produc* or coproduc* or co-design* or codesign* or input)).tw,kf | 2518 |
| 6 | (user* adj3 (participat* or involv* or engag* or partner* or collaborat* or contribut* or author* or coauthor* or co-author* or written or cowritten or co-written or coresearch* or co-research* or cocreat* or co-creat* or co-produc* or coproduc* or co-design* or codesign* or input)).tw,kf | 7004 |
| 7 | (citizen* adj3 (participat* or involv* or engag* or partner* or collaborat* or contribut* or author* or coauthor* or co-author* or written or cowritten or co-written or coresearch* or co-research* or cocreat* or co-creat* or co-produc* or coproduc* or co-design* or codesign* or input)).tw,kf | 1297 |
| 8 | (stakeholder* adj3 (participat* or involv* or engag* or partner* or collaborat* or contribut* or author* or coauthor* or co-author* or written or cowritten or co-written or coresearch* or co-research* or cocreat* or co-creat* or co-produc* or coproduc* or co-design* or codesign* or input)).tw,kf | 7541 |
| 9 | (parent* adj3 (participat* or involv* or engag* or partner* or collaborat* or contribut* or author* or coauthor* or co-author* or written or cowritten or co-written or coresearch* or co-research* or cocreat* or co-creat* or co-produc* or coproduc* or co-design* or codesign* or input)).tw,kf | 14224 |
| 10 | (caregiver* adj3 (participat* or involv* or engag* or partner* or collaborat* or contribut* or author* or coauthor* or co-author* or written or cowritten or co-written or coresearch* or co-research* or cocreat* or co-creat* or co-produc* or coproduc* or co-design* or codesign* or input)).tw,kf | 3644 |
| 11 | (community* adj3 (participat* or involv* or engag* or partner* or collaborat* or contribut* or author* or coauthor* or co-author* or written or cowritten or co-written or coresearch* or co-research* or cocreat* or co-creat* or co-produc* or coproduc* or co-design* or codesign* or input)).tw,kf | 24758 |
| 12 | ("as partners" adj4 research).tw,kf | 940 |
| 13 | ("knowledge users" adj3 research).tw,kf | 20 |
| 14 | ("service user" and (involvement or engagement)).tw,kf | 514 |
| 15 | ((engag* or participation) adj2 stakeholder?).tw,kf | 2136 |
| 16 | action research.tw,kf | 3866 |
| 17 | (coalition? adj3 health).tw,kf | 534 |
| 18 | community coalition?.tw,kf | 350 |
| 19 | community-based research.tw,kf | 757 |
| 20 | community-engaged research.tw,kf | 214 |
| 21 | disseminat* research.tw,kf | 407 |
| 22 | emancipatory research.tw,kf | 14 |
| 23 | engaged scholarship.tw,kf | 51 |
| 24 | (inclusive adj2 research).tw,kf | 127 |
| 25 | (knowledge transfer and exchange).tw,kf | 139 |
| 26 | participatory action research.tw,kf | 1128 |
| 27 | participatory design.tw,kf | 424 |
| 28 | participatory evaluation.tw,kf | 160 |
| 29 | participatory intervention?.tw,kf | 115 |
| 30 | participatory research.tw,kf | 3635 |
| 31 | patient and public involvement.tw,kf | 506 |
| 32 | (patient-centered adj2 research).tw,kf | 714 |
| 33 | patient-oriented research.tw,kf | 242 |
| 34 | (peer adj2 research).tw,kf | 835 |
| 35 | (research and ("peer led" or "public led" or "patient led" or "stakeholder led")).tw,kf | 296 |
| 36 | GRIPP.tw,kf | 25 |
| 37 | GRIPP2.tw,kf | 3 |
| 38 | (("mode 2" or "mode two" or "mode II") adj3 (knowledge or research)).tw,kf | 18 |
| 39 | linkage and exchange.tw,kf | 31 |
| 40 | integrated knowledge translation.tw,kf | 149 |
| 41 | collaborative research.tw,kf | 2753 |
| 42 | community-academic partnership.tw,kf | 197 |
| 43 | inclusive research.tw,kf | 79 |
| 44 | knowledge exchange.tw,kf | 542 |
| 45 | partnership research.tw,kf | 44 |
| 46 | patient empowerment.tw,kf | 1111 |
| 47 | patient activation.tw,kf | 824 |
| 48 | or/1-47 | 218614 |
| 49 | community participation/ | 16361 |
| 50 | patient participation/ | 23843 |
| 51 | community-based participatory research/ | 3821 |
| 52 | or/49-51 | 43342 |
| 53 | journal of patient experience.jn | 143 |
| 54 | research involvement & engagement.jn | 142 |
| 55 | or/53-54 | 285 |
| 56 | 48 or 52 or 55 | 248398 |
| 57 | scoping.tw,kw | 4453 |
| 58 | umbrella review*1.ti | 171 |
| 59 | review of reviews.ti | 203 |
| 60 | overview of reviews.ti | 55 |
| 61 | mapping review*1.ti | 65 |
| 62 | realist review*1.ti | 178 |
| 63 | rapid review*1.ti | 243 |
| 64 | metaanalys?s.ti | 657 |
| 65 | meta-analys?s.ti | 89867 |
| 66 | meta-review.ti | 105 |
| 67 | meta-synthesis.ti | 482 |
| 68 | knowledge synthesis.ti | 60 |
| 69 | (systematic adj2 review*1).ti | 105504 |
| 70 | (evidence adj2 synthesis).ti | 523 |
| 71 | systematic review.pt | 106499 |
| 72 | systematic reviews as topic/ | 2239 |
| 73 | meta-analysis.pt | 100866 |
| 74 | cochrane database of systematic reviews.jn | 14162 |
| 75 | or/57-74 | 216418 |
| 76 | 56 and 75 | 9841 |
| 77 | limit 76 to yr="2011 - Current" | 7735 |
