# Supplementary material for: Identification and Reporting of Patient and Public Partner Authorship on Knowledge Syntheses: Rapid Review
Source: J Particip Med. 2021 Jun 10;13(2):e27141. doi: 10.2196/27141 (PMC8235296; doi:10.2196/27141)
Supplement: Multimedia Appendix 2 [file jopm_v13i2e27141_app2.docx]

**Multimedia Appendix 2**

Modified search strategy for Ovid MEDLINE(R) and Epub Ahead of Print, In-Process & Other Non-Indexed Citations, Daily and Versions (run June 8, 2020)

| 1 | coresearcher*.ti,ab. |
| --- | --- |
| 2 | co-researcher*.ti,ab. |
| 3 | (reciprocity adj5 research).ti,ab. |
| 4 | (advisory councils adj5 research).ti,ab. |
| 5 | (citizen* adj5 research*).ti,ab. |
| 6 | (co-design* adj5 research).ti,ab. |
| 7 | (codesign* adj5 research).ti,ab. |
| 8 | (partnership* adj5 research).ti,ab. |
| 9 | peer research*.ti,ab. |
| 10 | (peer-led adj5 research).ti,ab. |
| 11 | public involvement.ti,ab. |
| 12 | "patient and family advisory".ti,ab. |
| 13 | "patient and family engagement".ti,ab. |
| 14 | "patient and public engagement".ti,ab. |
| 15 | "patient and public involvement".ti,ab. |
| 16 | patient empowerment.ti,ab. |
| 17 | patient engagement.ti,ab. |
| 18 | patient involvement.ti,ab. |
| 19 | patient participation.ti,ab. |
| 20 | patient research.ti,ab. |
| 21 | (patient-mediated adj5 research).ti,ab. |
| 22 | shared decision making.ti,ab. |
| 23 | shared decisionmaking.ti,ab. |
| 24 | (indigenous adj5 research).ti,ab. |
| 25 | torres strait islander people.ti,ab. |
| 26 | (carer engagement adj5 research).ti,ab. |
| 27 | community engagement.ti,ab. |
| 28 | community involvement.ti,ab. |
| 29 | community participation.ti,ab. |
| 30 | community partners.ti,ab. |
| 31 | (community-based adj5 research*).ti,ab. |
| 32 | (communitybased adj5 research*).ti,ab. |
| 33 | knowledge transfer.ti,ab. |
| 34 | knowledge translation.ti,ab. |
| 35 | knowledge users.ti,ab. |
| 36 | consumer involvement.ti,ab. |
| 37 | consumer participation.ti,ab. |
| 38 | cbpr.ti,ab. |
| 39 | (participatory adj5 research).ti,ab. |
| 40 | action research.ti,ab. |
| 41 | (stakeholder* adj5 research).ti,ab. |
| 42 | service user participation.ti,ab. |
| 43 | user involvement.ti,ab. |
| 44 | or/1-43 |
| 45 | critical appraisal.ti,ab. |
| 46 | integrative review.ti,ab. |
| 47 | knowledge synthes?s.ti,ab. |
| 48 | metaanalys?s.ti,ab. |
| 49 | meta-analys?s.ti,ab. |
| 50 | metanarrative.ti,ab. |
| 51 | meta-narrative.ti,ab. |
| 52 | metasynthes?s.ti,ab. |
| 53 | meta-synthes?s.ti,ab. |
| 54 | realist review.ti,ab. |
| 55 | (scoping adj2 review).ti,ab. |
| 56 | (systematic* adj2 review).ti,ab. |
| 57 | (systematic* adj2 search).ti,ab. |
| 58 | comprehensive search strategy.ti,ab. |
| 59 | embase psyc?info cinahl.ti,ab. |
| 60 | handsearch*.ti,ab. |
| 61 | medline embase.ti,ab. |
| 62 | prospero.ti,ab. |
| 63 | grey literature.ti,ab. |
| 64 | (data adj1 extract*).ti,ab. |
| 65 | prisma.ti,ab. |
| 66 | (review* adj2 independently).ti,ab. |
| 67 | search methods.ti,ab. |
| 68 | search strategy.ti,ab. |
| 69 | "titles and abstracts".ti,ab. |
| 70 | or/45-69 |
| 71 | 44 and 70 |
| 72 | (201905* or 201906* or 201907* or 201908* or 201909* or 201910* or 201911* or 201912* or 2020*).dt,ez,da. |
| 73 | 71 and 72 |
