# Supplementary material for: Identification and Reporting of Patient and Public Partner Authorship on Knowledge Syntheses: Rapid Review
Source: J Particip Med. 2021 Jun 10;13(2):e27141. doi: 10.2196/27141 (PMC8235296; doi:10.2196/27141)
Supplement: Multimedia Appendix 3 [file jopm_v13i2e27141_app3.docx]

**Multimedia Appendix 3**

Location of reporting about patient and public partners, and their roles when specified. When insufficient information was provided to connect the patient or partner role with any of the 12 review stages in the ACTIVE Framework, the authors have noted *unclear*.

| **Study** | **Where in article partner author(s) roles described** | **Stages of review partner author(s) contributed to** |
| --- | --- | --- |
| Brett J (2014a)[37] | Acknowledgments: Author Contributions | unclear |
| Brett J (2014b)[38] | Methods | plan methods, analyze data |
| Aslakson R [39] | n/a | unclear |
| Jones E (2015)[40] | Abstract, Methods, Discussion | develop question, develop search, select studies, analyze data, write and publish review |
| Whitton M (2015)[41] | Methods, Contributions | develop question, write and publish protocol, select studies, collect data, analyze data, write and publish review, knowledge translation and impact |
| Garvelink M (2016) [42] | n/a | unclear |
| Morley R (2016)[20] | Contributions | write and publish review |
| Souleymanov R (2016)[43] | Methods, Contributions | develop question, select studies, collect data, analyze data, write and publish review |
| Clarkson P (2017)[34] | Acknowledgements | unclear |
| Kronenberg C (2017)[44] | n/a | unclear |
| Bethell J (2018)[45] | Methods | knowledge translation |
| Crocker J (2018)[46] | Methods: Patient and Public Involvement | unclear |
| Evans D (2018)[35] | Methods: Patient and Public Involvement | unclear |
| Fergusson D (2018)[11] | Authors' Contributions | unclear |
| Jennings H (2018)[47] | Contributions | interpret findings, knowledge translation and impact |
| Jorgensen C (2018)[48] | n/a | unclear |
| Pollock A (2018)[49] | Abstract, Methods | write and publish protocol, develop search, interpret findings, write and publish review |
| Price A (2018)[50] | Methods, Results | select studies, collect data, analyze data, interpret findings, write and publish review |
| Baines R  (2019)[51] | Methods | unclear |
| Evans C (2019)[52] | Methods: Patient and Public Involvement | unclear |
| Gonzalez A (2019)[53] | Methods: Patient and Public Involvement | write and publish review |
| Greenhalgh T (2019)[4] | n/a | unclear |
| McCarron T (2019)[54] | Methods, Discussion, Contributions | develop question, write and publish protocol, develop search, select studies, analyze data, interpret findings, write and publish review |
| McGrath M (2019)[55] | n/a | unclear |
| Moore D (2019)[56] | End User Involvement, Contributions, Appendix | develop question, plan methods, develop search, select studies, analyze data, interpret findings, knowledge translation and impact, write and publish review |
| Oldfield (2019)[57] | Abstract, Methods, Results, Discussion | plan methods, develop search, interpret findings |
| Planner C (2019)[58] | Discussion, Contributions | interpret findings, write and publish review |
| Scholz B (2019)[59] | n/a | unclear |
| Sherriff N (2019)[36] | Abstract, Introduction, Methods, Limitations | develop question, plan methods, develop search, select studies, collect data |
| Bird M (2020)[60] | n/a | unclear |
| Brush B (2020)[61] | n/a | unclear |
| Gordon M (2020)[62] | Patient/Service User Involvement, Discussion | unclear |
| Graham L (2020)[63] | Methods | develop question, interpret findings, write and publish review |
| Hoekstra F (2020)[14] | Methods: Engagement of Stakeholders in the Review | develop question, analyze data, interpret findings, knowledge translation and impact |
| Hung L (2020)[64] | Methods: Search Strategy | unclear |
| Maidment I (2020)[65] | Contributions | analyze data, write and publish review |
| Arnstein L (2020)[15] | Abstract, Introduction, Results | write and publish protocol, interpret findings, knowledge translation and impact |
